# Exploring species-level infant gut bacterial biodiversity by meta-analysis and formulation of an optimized cultivation medium

**DOI:** 10.1038/s41522-022-00349-1

**Published:** 2022-10-31

**Authors:** Giulia Alessandri, Federico Fontana, Leonardo Mancabelli, Gabriele Andrea Lugli, Chiara Tarracchini, Chiara Argentini, Giulia Longhi, Alice Viappiani, Christian Milani, Francesca Turroni, Douwe van Sinderen, Marco Ventura

**Affiliations:** 1 Laboratory of Probiogenomics, Department of Chemistry, Life Sciences, and Environmental Sustainability, University of Parma, Parma, Italy; 2 GenProbio srl, Parma, Italy; 3 Microbiome Research Hub, University of Parma, Parma, Italy; 4 APC Microbiome Institute and School of Microbiology, Bioscience Institute, National University of Ireland, Cork, Ireland

**Keywords:** Applied microbiology, Environmental microbiology

## Abstract

In vitro gut cultivation models provide host-uncoupled, fast, and cost-efficient solutions to investigate the effects of intrinsic and extrinsic factors impacting on both composition and functionality of the intestinal microbial ecosystem. However, to ensure the maintenance and survival of gut microbial players and preserve their functions, these systems require close monitoring of several variables, including oxygen concentration, pH, and temperature, as well as the use of a culture medium satisfying the microbial nutritional requirements. In this context, in order to identify the macro- and micro-nutrients necessary for in vitro cultivation of the infant gut microbiota, a meta-analysis based on 1669 publicly available shotgun metagenomic samples corresponding to fecal samples of healthy, full-term infants aged from a few days to three years was performed to define the predominant species characterizing the “infant-like” gut microbial ecosystem. A subsequent comparison of growth performances was made using infant fecal samples that contained the most abundant bacterial taxa of the infant gut microbiota, when cultivated on 18 different culture media. This growth analysis was performed by means of flow cytometry-based bacterial cell enumeration and shallow shotgun sequencing, which allowed the formulation of an optimized growth medium, i.e., Infant Gut Super Medium (IGSM), which maintains and sustains the infant gut microbial biodiversity under in vitro growth conditions. Furthermore, this formulation was used to evaluate the in vitro effect of two drugs commonly used in pediatrics, i.e., acetaminophen and simethicone, on the taxonomic composition of the infant gut microbiota.

## Introduction

The human gastrointestinal tract (GIT) is inhabited by an extraordinary complex and dynamic microbial consortium, collectively referred to as the gut microbiota, which plays a crucial role in defining the host health status by influencing and modulating physiological, metabolic, and immunological functionalities^1,2^. Starting from birth, when the very first assembly of gut microbiota occurs due to the exposure to maternal and environmental bacterial communities, the following three years of life are of vital importance for the subsequent development and maturation of this delicate microbial ecosystem^3–5^. Indeed, during this period, the gut microbiota is continuously subject to modifications as driven by positive and negative interactions between key microbial players, which are influenced by several perinatal and postnatal factors, including mode of delivery, feeding type, antibiotic exposure, post-weaning diet, and host genetics^6,7^. Despite the apparent instability of the infant gut microbiota, this early stage of life represents a window of opportunity for the establishment of an interactive host-bacteria dialogue on which the development of both the host immune system and gut homeostasis rely^8,9^. Consequently, interactions that occur between the host and its intestinal microbial community during infancy are not only critical for immediate or transient health effects, but they have also been implicated in long-term health outcomes^8^. For this reason, substantial efforts have been made to unveil the assembly and development of the infant gut microbiota^10–14^. In this context, recent advances in high-throughput molecular analyses, with the advent of multiple ‘-omics’ technologies, have generated in depth insights into the ecology of the infant gut microbial community^15–18^. However, omics-based studies are limited to observational and/or predictive investigations, and do not provide direct experimental proof to uncover microbe-microbe or microbe-host interactions. In this context, in vitro gut cultivation models represent an appropriate, powerful, and yet host-uncoupled tool to study the effects of intrinsic and extrinsic factors affecting both composition and functionality of the intestinal microbial population, while avoiding any ethical concerns related to in vivo studies^19–21^. Indeed, using a growth medium able to reproduce the intestinal environment coupled with a strict control of several variables, including oxygen concentration, pH and temperature, in vitro cultivation models may allow stabilization of the gut microbiota over time, preserving dominant microbial groups and associated metabolic activities^22^. However, although the composition of the growth medium is of critical importance to ensure growth of the various members of the human gut microbiota, most of the currently available formulations have been devised for adult gut microbiota cultivation, thus overlooking the peculiar characteristics of the infant gut microbiota and its related environment^23^.

In this context, with the aim of formulating a specific growth medium able to reliably perform in vitro cultivation of the infant gut microbiota, the taxonomic composition of the infant intestinal ecosystem was assessed by selecting 1669 publicly available shotgun metagenomic samples corresponding to fecal samples of healthy, full-term infants aged from one month to three years of life. The re-analysis of these samples not only allowed the definition of major microbial players of the infant gut microbiota but also identified microbial taxonomic patterns of the infant gut microbiota, revealing the existence of distinct microbial assemblies, defined here as species-level Infant Gut Community State Types (sIGCSTs). Based on these data, infant fecal samples representing the identified sIGCSTs were cultivated on different culture media, generally employed for the in vitro cultivation of the human gut microbiota, to identify those specific medium components supporting growth of infant gut bacteria. Subsequently, an optimized growth medium, here referred to as the Infant Gut Super Medium (IGSM), was formulated and assessed for its ability to support in vitro growth of common elements of the infant gut microbiota. Furthermore, IGSM was applied to cultivate the infant gut microbiota under in vitro settings and to investigate the effects of drugs commonly used in pediatric treatments, i.e., acetaminophen and simethicone, on infant gut microbiota taxonomic composition.

## Results

### Selection of public datasets

To formulate an ad hoc culture medium suitable for in vitro cultivation of bacterial taxa that are characteristic of the infant gut microbiota, a meta-analysis was performed aimed at defining the taxonomic composition of the infant intestinal microbial ecosystem based on publicly available shotgun metagenomic datasets. For this purpose, an in-depth literature search was carried out to retrieve shotgun metagenomic datasets based on Illumina sequencing technology corresponding to fecal samples from healthy, full-term infants that ranged in age from one month to 36 months, since the transition from an infant- to an adult-like gut microbiota is thought to have occurred by the time the infant reaches three years of age^4,9,17,24,25^. Moreover, in case of longitudinal studies, only one sample per infant was considered to avoid redundant samples, while for studies involving the administration of drugs, prebiotics and/or probiotics, only infant fecal samples belonging to the control group were selected. This allowed for a collection of 2411 publicly available samples, selected from 17 cohorts, and covering different geographical areas. Furthermore, based on the assumption that the infant gut microbiota is highly variable and dynamic during the first three years of life, the selected samples were divided into three different age-based groups, i.e., group 1-6 M (from 30 days to 6 months of life), group 6-12 M (between 6 months and one year of life), and group 12-36 M (from one to three years of age), as previously described^9,17^. This grouping resulted in a total of 1328, 372 and 711 samples corresponding to the 1-6 M, 6-12 M and 12-36 M groups, respectively. Details regarding the selection of public datasets are discussed in further detail in the supplementary text.

### Meta-analysis of the infant gut bacterial community and identification of the dominant bacterial species

To provide an in-depth evaluation of the bacterial species typical of the human gut microbiota during infancy and to standardize the bioinformatic pipeline, 100,000 reads per each sample of the selected 2411 publicly available samples were re-analyzed by means of the recently published METAnnotatorX2 software pipeline, which is specifically designed to process shotgun metagenomics data sets^26^. Specifically, 100,000 reads were processed for each sample as they correspond to the proposed default number of reads required to achieve a reliable taxonomic profile of bacterial species present at >0.5% relative abundance, which is a cut-off that was selected to provide robustness to the taxonomical analyses in order to eliminate bacterial species with severely reduced abundance, and compatible with shallow shotgun metagenomics applications, as previously described^26^. Furthermore, despite the fact that the NCBI-based RefSeq database does not include Metagenome Assembled Genomes (MAGs) and that it is not fully representative of bacterial species typical of non-Westernized populations, the RefSeq was employed as the reference database for the METAnnotatorX2 pipeline-based analysis^26,27^. Indeed, the use of the RefSeq database allowed us to exclusively use whole-genome shotgun assemblies with high quality annotations making the analysis more accurate and robust^26,28^.

Based on the obtained species-level taxonomic profiles, a β-diversity analysis was performed through a Principal Coordinate Analysis (PCoA) representation to eliminate any outliers. Based on the latter analysis and the above-mentioned exclusion criteria, a total of 1669 samples were retained to define the principal bacterial players of the infant gut microbiota (Table 1 and Supplementary Table 1). After quality checks and removing *Homo sapiens* reads, a total of 106,519,353 reads were taxonomically classified with an average of 63,822 ± 15,018 reads per sample (Supplementary Table 2). Taxonomic reconstruction of the bacterial community of each analyzed sample is reported in Supplementary Material (Supplementary Table 3).

**Table 1:** Metadata associated to the fecal samples included in this study.

Study (PMID)	Bioproject	Number of samples	Age (days) (mean ± st.dev)	Nation		Tecnology
26308884	PRJEB6456	200	240 ± 120.30	Sweden	Illumina	
27133167	PRJNA290380	395	545.48 ± 293.55	Finland/Estonia/Russia	Illumina	
27255079	PRJNA345144	125	362.16 ± 313.33	New Zealand	Illumina	
28144631	PRJNA339914	5	90 ± 0	Italy	Illumina	
32284564	PRJNA557731	86	30 ± 0	Africa-America	Illumina	
26231653	PRJNA287207	2	90 ± 0	Italy	Illumina	
30001517	PRJNA475246	60	90 ± 0	USA	Illumina	
34278055	PRJNA542703	14	45.36 ± 0.50	USA	Illumina	
25405552	PRJEB32135	66	249.55 ± 133.77	Canada	Illumina	
27583441	PRJEB12669	14	544.29 ± 386.50	China	Illumina	
32461640	PRJNA524703	307	156.12 ± 165.50	Urban United States cohort of African American infants	Illumina	
31832638	PRJNA549787	56	113.34 ± 59.48	South Africa	Illumina	
30374198	PRJNA473126	57	234.35 ± 16.54	USA	Illumina	
30126456	PRJNA422569	3	730 ± 365	Italy	Illumina	
30001516	PRJNA352475	14	120 ± 0	Italy	Illumina	
28112736	PRJNA322188	32	43.60 ± 0	USA	Illumina	
26578308	PRJEB24771	233	183.46 ± 68.88	Malawi	Illumina	

**Fig. 1:**
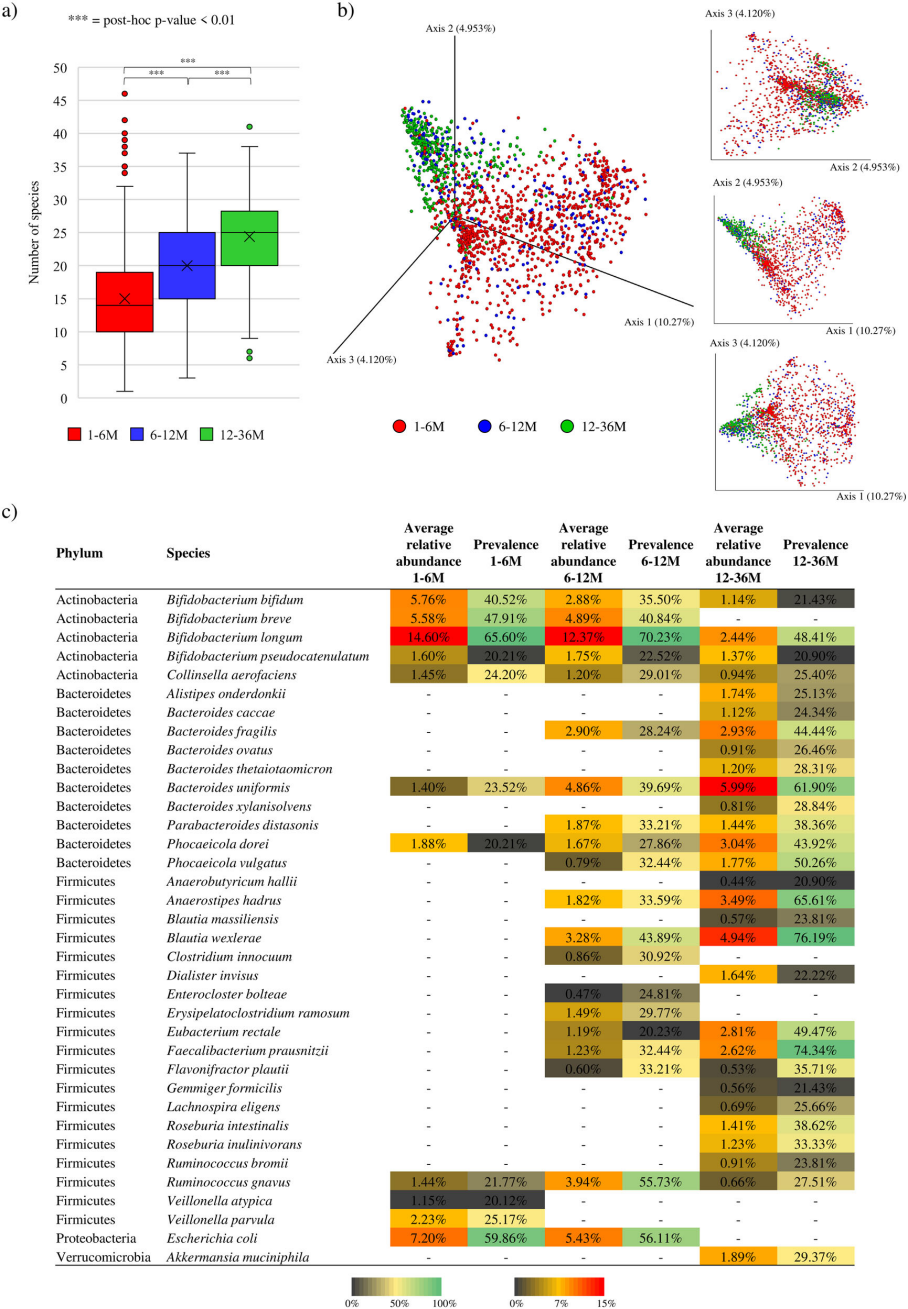
Evaluation of the bacterial diversity and taxonomic composition of healthy infant fecal samples over time. Panel **a** shows the box and whisker plot of the calculated species-richness based on the number of bacterial species observed in the three age group. The x-axis reports the three considered age groups, while the y-axis shows the number of bacterial species. Boxes are determined by the 25^th^ and 75^th^ percentiles. The whiskers are determined by the maximum and minimum values and correspond to the box extreme values. Lines inside the boxes represent the average, while crosses correspond to the median. Panel **b** displays both the two- and three-dimensional Bray-Curtis dissimilarity index-based PCoA of the 1669 selected infant fecal samples subdivided by age groups. Panel **c** reports the average relative abundance and prevalence of bacterial species corresponding to the core and accessory gut microbiota of each age group.

As expected from already published single cohort-based and/or longitudinally studies, species richness analysis revealed a progressive and statistically significant increment in the average number of species across groups that positively correlated with age (ANOVA post-hoc *p* value < 0.01) (Fig. 1)^29^. Moreover, a Bray-Curtis dissimilarity-based beta-diversity analysis, represented through PCoA, highlighted clear compositional differences between samples belonging to 1-6 M or 6-12 M, when compared to those of the 12-36 M group (Fig. 1). In this context, ANOSIM statistics revealed statistically significant differences between the fecal microbiota composition of infants belonging to the 1-6 M and 6-12 M groups with respect to the already weaned group (*p*-value <0.01 in both cases), emphasizing the pivotal role played by the transition to an exclusively solid diet on influencing the taxonomic composition of the infant gut microbiota. In addition, beta-diversity analysis revealed a highly variable bacterial composition within the 1-6 M group, while the intestinal community within the 6-12 M and 12-36 M seemed to gradually stabilize (Fig. 1), an observation that correlates with the already demonstrated high instability and inter-individual variability of gut microbiota during the very early stages of life^29,30^. Additional details can be found in the Supplementary Text.

Furthermore, taxonomic profiles corresponding to the 1669 selected shotgun metagenomic samples were scrutinized to identify the most representative bacterial species of the 1-6 M, 6-12 M and 12-36 M age groups, allowing the reconstruction of dominant bacterial species of the infant gut microbiota, i.e., the “core” infant gut microbiota^31^. Specifically, this “core” community was defined by selecting those bacterial species occurring with a prevalence of >40% among the assessed samples of a specific age group, while bacterial taxa showing a prevalence ranging from 20% to 40% were used to identify infant gut accessory bacterial taxa. These cut-off values were adjusted from those suggested in previous literature based on genus-level data to obtain more permissive prevalence ranges, considering the high variability of the infant gut microbiota, the wide range of geographical locations from which the considered samples derived, and the higher resolution offered by species-level accuracy^17,32–35^. Based on these parameters, the “core” gut microbiota of the 1-6 M group was found to consist of only four different species, i.e., *Bifidobacterium bifidum, Bifidobacterium breve, Bifidobacterium longum*, and *Escherichia coli*. The same taxa, except for *Bifidobacterium bifidum*, were also observed to be part of the “core” gut microbiota of the 6-12 M group together with other two additional species, i.e., *Blautia wexlerae* and *Ruminococcus gnavus*. Conversely, the core intestinal community of the already weaned infants (12-36 M group) was characterized by a higher microbial complexity, typical of an adult-like gut microbiota (Fig. 1)^9,18^, including *Anaerostipes hadrus*, *Bacteroides fragilis, Bacteroides uniformis*, *Bifidobacterium longum, Blautia wexlerae*, *Eubacterium rectale*, and *Faecalibacterium prausnitzii*, coupled with two other species formerly belonging to the genus *Bacteroides*, but recently reclassified as members of the genus *Phocaeicola*, i.e. *Phocaeicola dorei* and *Phocaeicola vulgatus* (Fig. 1)^36^. Furthermore, this analysis revealed that the increasing number of “core” species across the 12-36 M age group corresponds to a progressive increment in the number of species forming the so-called infant accessory gut microbiota. Indeed, while only seven taxonomically annotated species were included in the accessory microbiota of the 1-6 M group, 15 and 20 bacterial species represented the accessory gut microbial community of the 6-12 M and 12-36 M groups, respectively (Fig. 1). This reinforces the notion that weaning signals the diversification of the infant gut microbiota towards an adult-like gut microbiota with a gradual expansion of the species number.

The “core” microbiota assessed per each age group allowed the identification of the most prevalent species across all samples belonging to a single age group regardless of diet or geographical origin. In this context, to make the analysis more accurate, since diet and geographical origin have been indicated as two main factors deeply involved in influencing gut microbiota composition, the “core” microbiota for each age group was also assessed introducing the geographical locations of infants to which fecal samples belong as variable (Supplementary Table 4). Additional information is discussed in detail in the supplementary text.

### Identification of species-level Infant Gut Community State Types (sIGCSTs)

**Fig. 2:**
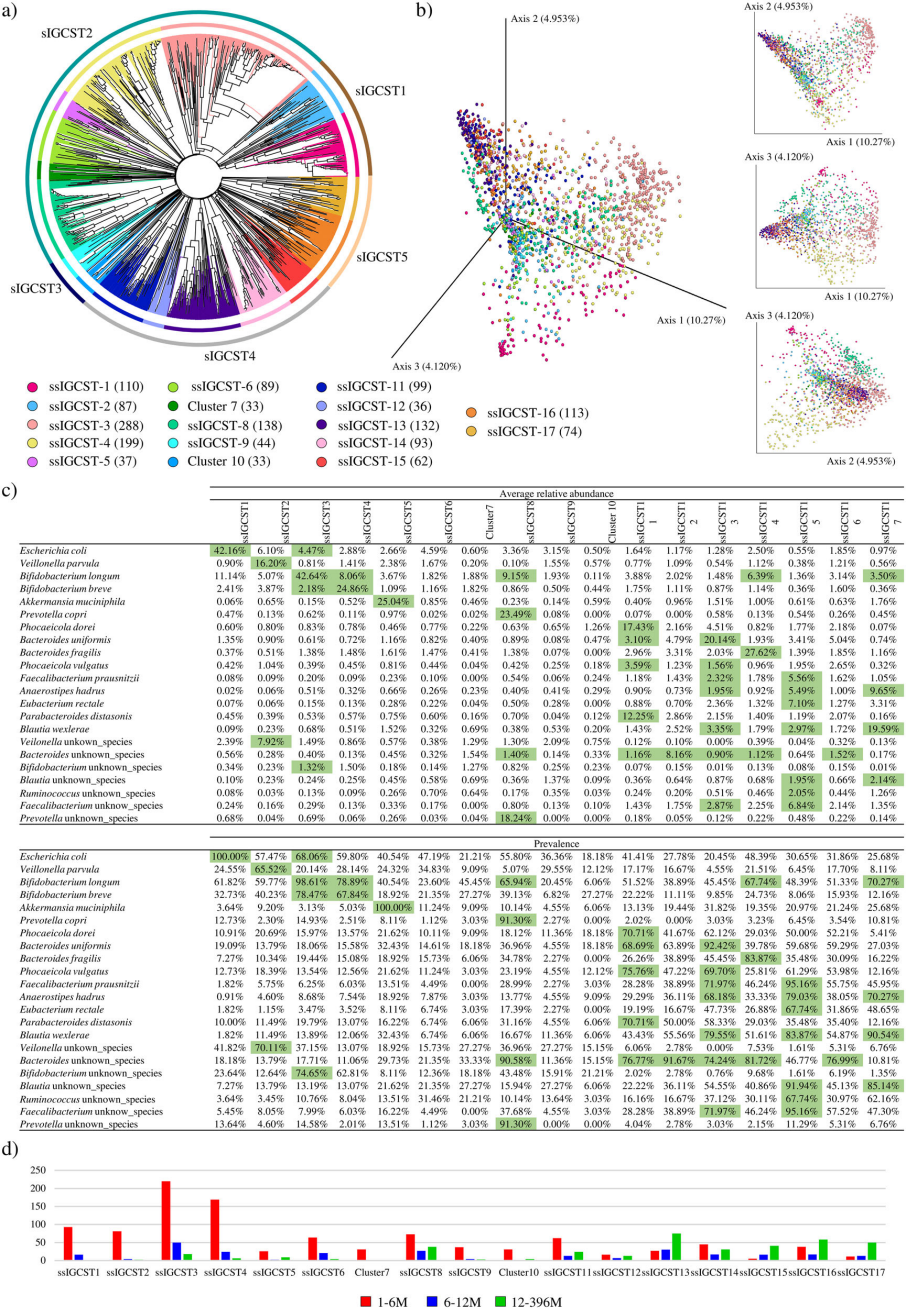
Identification of species-level infant gut community state types (sIGCSTs). Panel **a** reports a circular cladogram generated through a hierarchical clustering (HCL) analysis based on selected publicly available shotgun metagenomes related to fecal samples of healthy infants aged from a few days to three years. The outer circle around the cladogram divides samples according to the identified sIGCSTs, while the inner circles provide a subdivision of fecal samples based on the defined sub-sIGCSTs. Panel **b** shows the three- and two-dimensional Principal Coordinate Analysis (PCoA) based on the same samples used for the HCL by coloring samples according to the ssIGCST they belong to. Color legend for panel **a** and **b** reports the cluster name and the number of infant fecal samples per each identified ssIGCST. Panel **c** displays the average abundance and prevalence of bacteria specifying each identified ssIGCSTs. Panel **d** depicts the distribution of samples for each ssIGCST divided by age groups.

Although the reconstruction of a “core” infant gut microbiota allows the identification of the most prevalent bacterial species across a sample cohort, it does not identify possible distinct motifs in the overall community composition profile, thereby ignoring the presence of highly abundant and representative bacterial species in a subset of samples^37–40^. Therefore, in order to provide stratification of the selected samples into compositional patterns, the 1669 shotgun-based microbial profiles were subjected to two unsupervised analyses, i.e., Prediction Strength and Silhouette Width^41^, allowing the prediction of the minimal number of clusters necessary to divide samples into distinct community state types. Specifically, the unsupervised Prediction Strength analysis and the Silhouette-based method identified an optimal number of four and five clusters, respectively (Supplementary Figure 1). Based on these data, pre-setting the number of clusters to be identified to five, a Hierarchical CLustering analysis (HCL) based on taxonomic profiles at species level of each enrolled sample was performed, allowing the division of samples into five different groups, here referred as species-level Infant Gut Community State Types (sIGCSTs) (Fig. 2). In depth insights into the identified sIGCSTs revealed that four clusters are dominated by a single bacterial species with a high prevalence (>65%), i.e., *E. coli* (sIGCST1), *B. longum* (sIGCST2), *B. uniformis* (sIGCST4), and *B. wexlerae* (sIGCST5), while sIGCST3 did not show any dominant bacterial taxon (Supplementary Table 5). In this context, although the sIGCSTs prevalent species corresponded to some of the bacterial taxa above-defined as “core” gut microbiota-characterizing species, most of the latter failed to be represented by the identified sIGCSTs. Therefore, a supervised cluster analysis based on a Bray-Curtis dissimilarity matrix-related Hierarchical Clustering (Supplementary Table 6) and supported by 3D Bray-Curtis PCoA (Fig. 2) was performed to implement and biologically verify the results of the unsupervised approaches, as previously described^42^, and to identify putative sub-sIGCSTs (ssIGCSTs) representing a continuous spectrum of variability within the five primary sIGCSTs. In detail, to be considered as a putative ssIGST, each cluster had to include at least 20 samples (approximately 1% of total samples), while the ssIGCST representative bacterial species had to possess a prevalence higher than 65%. Interestingly, this analysis allowed the subdivision of the five sIGCSTs into 17 putative sub-clusters with sIGCST1, sIGCST3, and sIGCST5 split into two sub-clusters each, while sIGCST2 and sIGCST4 were divided into six and five sub-clusters, respectively (Fig. 2). However, the descriptive analysis based on two-way frequency tables revealed that more than 90% of clusters 7 and 10 were from a single geographic origin/dataset (Supplementary Table 7), thus these clusters were not considered as real ssIGCSTs. Further information about the results obtained for cluster validation based on two-way frequency tables has been reported in Supplementary text. According to these parameters, only 13 out of the 17 identified sub-clusters were shown to be effectively dominated by a minimum of one to a maximum of six predominant bacterial species (Fig. 2). Interestingly, most of the latter correspond to the abovementioned “core” infant gut microbiota-characterizing taxa, thus confirming the evolutionary adaptation of these species to the infant intestine. However, the HCL analysis also allowed the identification of an additional annotated species, previously not classified as “core” taxa, i.e., *Prevotella copri*, as representative of specific sub-cluster (Fig. 2), suggesting its ecological relevance in the assembly and development of the infant gut microbiota.

In depth insights into the assessed ssIGCSTs revealed that four sub-clusters were mainly represented by fecal samples of the 1-6 M, allowing the identification of *E. coli*, *V. parvula*, *B. longum*, and *B. breve* as the principal bacterial species that characterize the gut microbiota of pre-weaning infants (Fig. 2 and Supplementary Table 8). Conversely, ssIGCST13 and ssIGCST17 were preponderant among fecal samples belonging to the 12-36 M infant group, suggesting that *A. hadrus, B. uniformis*, *F. prausnitzii*, *P. vulgatus*, and *B. wexlerae* are five dominant species of an infant intestinal community developing into an adult-like gut microbiota (Fig. 2 and Supplementary Table 8). At the same time, the majority of the remaining 12-36 M samples were included in either of four clusters, all dominated by members of the phylum Bacteroidota, i.e., sIGCST8, 11, 14, and 16, as well as in two clusters characterized by a higher bacterial complexity with various dominant taxa, i.e., ssIGCST16 and 17. These findings underscore that, when developing and maturing into an adult-like gut microbiota, the infant gut community undergoes a transition with a decrease in the relative abundance of species belonging to bacterial genera typical of the pre-weaning phase (i.e. *Bifidobacterium* and *Escherichia*) with a parallel increase in taxa belonging to bacterial genera typical of an adult gut community (*Bacteroides*/*Phocaeicola* and *Prevotella*)^40,43^. Furthermore, these observations also highlight that the infant gut microbiota experiences an increase in bacterial biodiversity with, in certain cases, a concomitant decrease in the abundance of dominant species (Fig. 2).

### Infant fecal sample growth performances in different culture media and prediction of macronutrient requirements for ssIGCST communities

In vitro cultivation models have been developed as cost- and time-efficient, yet powerful tools to study the effects of intrinsic and extrinsic factors on both composition and functionality of the human gut microbiota^19,44^. Notably, while in vitro batch cultures do not involve human or animal hosts, thus preventing ethical concerns related to in vivo studies, they nonetheless require careful consideration of culture medium formulation to meet the nutritional needs of intestinal microorganisms and preserve/maintain the microbial players^19,21,45^. However, although currently used bacterial growth media for in vitro cultivation of the human gut microbial community are generally composed of complex and chemically undefined components, they differ in formulation and component concentrations^21,46–49^.

In this context, in order to identify which components are necessary to obtain a successful in vitro cultivation of the infant gut microbiota, five fecal samples of infants aged between four and 29 months and comprising the identified ssIGCST-dominant species, were grown for 24 h in duplicate on 18 different commercially available culture media generally used for human gut microbiota cultivation, following the MiPro model (Supplementary Table 9 and Supplementary Table 10)^21^. Taxonomic composition of each biological replicate at 24 h, coupled with two replicates obtained at 0 h (immediately after inoculation), was assessed through a shallow shotgun metagenomics approach. DNA sequencing generated a total of 15,198,683 reads with an average of 79,993 reads per sample, reduced to 9,982,665 total reads with an average of 52,540 reads per sample after quality filtering (Supplementary Table 11). Furthermore, to obtain a comprehensive biological interpretation of the analyzed culture microbiome complexity, a quantitative microbiome profiling assay was performed by means of flow cytometry to enumerate microbial cells of each biological replicate and culture condition, as previously described^50^. The obtained cell counts were subsequently employed to normalize shallow shotgun sequencing data and transform relative metagenomic data into absolute abundances.

Notably, bacterial cell number of the 24 h cultures exceeded the average of the inoculum cell number from 1.23- to 42.65-fold, confirming that all tested culture media support the growth of the infant gut microbial community (Supplementary Figure 2). However, high variability in dominant- as well as accessory-ssIGCST species growth performances was observed depending on culture medium (Supplementary Figure 2), suggesting that the success of culturing the infant gut microbial community is severely affected by the particular growth medium formulation used. In order to evaluate if and how a specific medium component impacts on the growth of ssIGCST-specifying bacterial species, a Pearson correlation index-based co-variance analysis was performed (Supplementary Table 12). Specifically, bacterial taxa typical of the 1-6 M group intestinal microbiota together with certain bacterial species characterizing the other two age groups positively correlated with at least one complex plant-derived glycan, such as inulin, pectin, arabinogalactan, maltodextrin, guar gum or xylan (Supplementary Table 12). Notably, members of the genus *Bifidobacterium* are skilled degraders of a wide range of plant-derived carbohydrates due to the presence, in their genetic arsenal, of several genes dedicated to this activity^51–53^, while they also participate in intra- and inter-genus cross-feeding behaviour^25,54–56^. Similarly, members of the genera *Anaerostipes*, *Faecalibacterium, Prevotella* and *Ruminococcus* are known for their ability to utilize different complex sugars as energy source^57–60^. Furthermore, the presence of a host-derived complex glycan, i.e., mucin, significantly sustained growth of *Bifidobacterium bifidum*, *Bifidobacterium longum* and *R. gnavus*, three bacterial taxa possessing, in their genetic repertoire, genes involved in the degradation of this host-related carbohydrate (Supplementary Table 12)^52,61–66^. Moreover, considering simple carbon sources, *Blautia wexlerae*, *B. bifidum*, *B. longum* and *E. coli* positively correlated with lactose, while *Bacteroides fragilis* and *V. parvula* with glucose. Conversely, xylo-oligosaccharides, cellobiose and maltose do not significantly support growth of any of the ssIGCST-identifying species (Supplementary Table 12).

In addition to chemically defined carbon sources, growth media generally used for the in vitro cultivation of gut microbiota contain numerous chemically undefined substances acting as suppliers of multiple organic and inorganic nutrients. Specifically, tryptone, casein, peptone and yeast extract were shown to elicit positive correlations with various dominant bacterial species of the infant microbial gut community, suggesting that these undefined components are necessary to ensure the survival and proliferation of the infant gut bacterial players (Supplementary Table 12). Furthermore, *Bacteroides fragilis* and *V. parvula* (and in general all the species belonging to the genus *Veillonella*) exhibited statistically significant positive correlations only with the various undefined components of medium 9 (GAM broth) (Supplementary Table 10 and Supplementary Table 12), as previously described for *V. parvula*, a bacterial species that, despite the presence of complex plant-derived carbohydrates in the culture medium, seems to require only undefined substances for its growth and proliferation^67^.

Remarkably, screening of the accessory bacteria constituting the predicted ssIGCST confirmed the relevance of the range of macronutrients required for the growth of dominant taxa (Supplementary Table 12). Altogether, these data allowed the identification of 16 macronutrients, which were used to formulate an optimized growth medium for the in vitro cultivation of infant gut microbial communities, here referred to as Infant Gut Super Medium (IGSM).

### Prediction of micronutrient requirements to sustain the growth of ssIGCST communities

Beyond macronutrients, bacterial growth requires micronutrients, generally represented by organic and inorganic salts as well as vitamins. Specifically, two inorganic salts widely exploited in culture medium formulation, i.e., sodium chloride (NaCl) and potassium chloride (KCl), showed a positive correlation with most of ssIGCST-dominant species (Supplementary Table 12), probably due to their involvement in culture medium osmolarity maintenance^68,69^. Furthermore, traces of other mineral salts seemed to sustain the growth of various bacterial species typical of the infant gut microbiota, including both dominant and non-dominant taxa (Supplementary Table 12), suggesting that several bacterial gut colonizers need the supply of different metals for their proliferation. Similarly, most of the ssIGCST-dominant bacterial species positively correlated with vitamin supplementation (Supplementary Table 12), thus highlighting the relevant role played by these trace elements in sustaining infant gut microbiota metabolism^70^.

In addition to mineral salts and vitamins, other growth factors may be vital to boost proliferation of certain infant gut microbiota-associated bacteria. In this context, the presence of L-cysteine significantly correlated with a range of bacteria, including *Bifidobacterium* spp. and *Ruminococcus gnavus*. Conversely, as expected for their ability to inhibit the growth of a wide spectrum of bacteria, only few positive correlation trends were observed between commercialized bile salt mixture and infant gut microbiota accessory species, while ssIGCST-dominant taxa negatively correlated with these compounds (Supplementary Table 12)^70^. However, an in-depth evaluation of these compounds revealed that the replacement of commercialized bile salt mixture with two defined major human primary bile acids, i.e., sodium cholate and sodium chenodeoxycholate, resulted in significantly positive correlations also with dominant taxa, thus corroborating a previous finding according to which this replacement was effective in maintaining the in vitro microbiome composition^21^.

Screening for micronutrients pivotal for the in vitro cultivation of ssIGCST resulted in the identification of 27 compounds which were employed, in addition to the above-defined macronutrients, to develop the IGS medium.

### Validation of the optimized culture medium IGSM for the in vitro cultivation of the infant gut microbiota

**Fig. 3:**
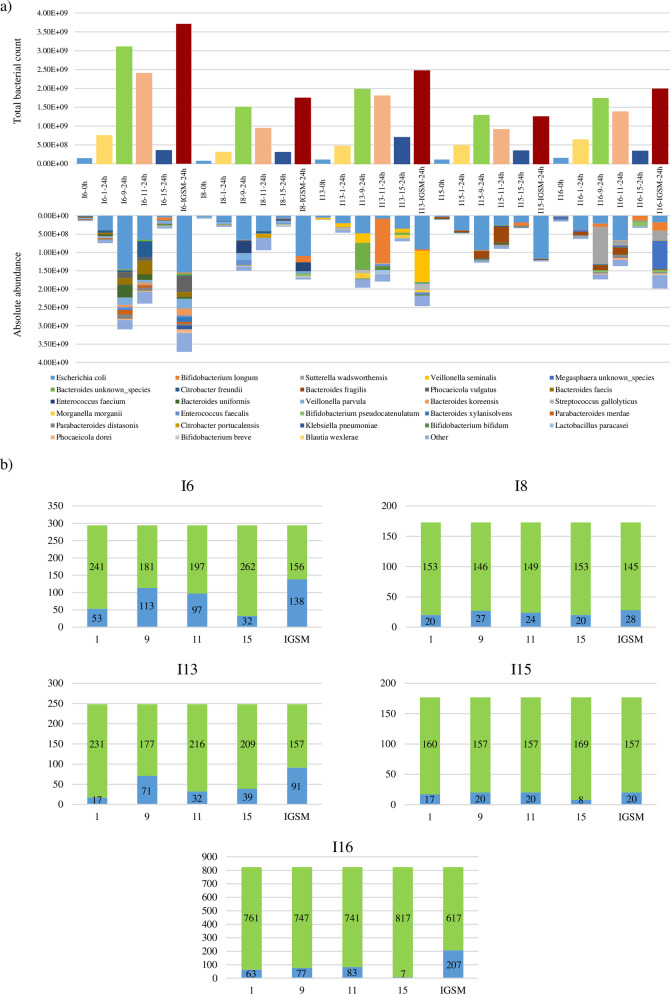
Validation of the IGSM formulation. Panel **a** depicts the flow cytometry-based bacterial counts and absolute abundance-based taxonomic profiles obtained by cultivating five infant fecal samples in four different culture media as well as in IGSM. Panel **b** reports the number of bacterial species showing a 10-fold increase in bacterial enumeration higher (light blue) or lower (green) than the inoculum bacterial count. All data are expressed as average of the two obtained replicates per each condition.

To evaluate the effectiveness of the IGSM, five fecal samples belonging to infants aged from one to six months and possessing the 1-6 M group ssIGCST-characterizing bacterial species were cultured for 24 h in duplicate in IGSM as well as in media 1, 9, 11 and 15 that, from the above-mentioned analysis, were shown to allow growth of the highest number of species present in the inoculum (Supplementary Table 10 and Supplementary Figure 2). After cultivation, growth performances of each infant fecal sample were assessed through flow cytometry-based bacterial cell enumeration. Interestingly, although an increase in bacterial cell number was recorded for all fecal samples per each tested culture medium, flow cytometry readouts related to 24 h cultivations exceeded by at least 10 times (when compared to the cell counts of the inoculum) for only three growth media, i.e., medium 9, 11 and IGSM (Fig. 3), except for I15 and I16 fecal samples whose growth in medium 11 resulted in an 8-fold increase. Furthermore, in depth insight into the latter three growth media revealed that the highest cell numbers were achieved for IGSM, with an increase ranging from 11.1- to 25.3-fold, against the maximum increase of 21.1- and 16.3-fold obtained in medium 9 and 11, respectively (Fig. 3). This indicates that the optimized growth medium formulation supports growth of the infant gut microbiota in terms of total bacterial count. However, such enhanced growth capacity of the infant gut microbial ecosystem by IGSM is not the only prerequisite for effectiveness of this new formulation. To achieve this goal, it is, indeed, also necessary that IGSM is not only able to sustain growth of the predominant species of the infant gut microbiota, but it should also ensure growth of many, if not all, of the accessory species. To assess this, each 24 h replicate and 0 h baseline samples were subjected to shallow shotgun sequencing resulting in a total of 2,224,237 reads, reduced to 1,657,573 reads with an average of 27,626 reads per sample after quality filtering (Supplementary Table 13). Furthermore, following taxonomic reconstruction, each obtained taxonomic profile was converted from relative to absolute abundance exploiting flow cytometry-based bacterial counts. Subsequently, in order to evaluate which of the five tested culture media was able to support growth of the highest number of species, only bacterial taxa showing an increased count in the 24 h samples that is higher than 10 times that of the inoculum, were considered. This cut-off was set to include only those bacterial species in the analysis that have undergone appreciable growth during the 24 h of cultivation and, at the same time, eliminating all those bacterial taxa whose presence in the 24 h samples may be attributable to the DNA of the inoculum. Interestingly, for each considered infant fecal sample, the highest number of species showing a 10-fold count increase with respect to the inoculum was achieved in IGSM (Fig. 3). This finding supports the notion that the optimized IGSM in vitro better maintains infant gut microbiota biodiversity when compared to other tested growth media. Altogether, these data indicate the effectiveness and robustness of this newly formulated growth medium in supporting growth and maintenance of most relevant bacterial players of the infant gut microbiota.

### Evaluation of the effects of drugs on the infant gut microbiota under in vitro settings

To evaluate the impact of certain drugs on the infant gut bacterial community, a 24 h in vitro cultivation of feces collected from the five infants whose stool were used for the IGSM validation, was performed in IGSM in the presence of two pharmacological compounds generally exploited in pediatrics, i.e., acetaminophen and simethicone. Feces were freshly recollected three weeks after the first collection and checked for the presence of the ssIGCST-characterizing species through shallow shotgun sequencing. Specifically, while acetaminophen is an analgesic/antipyretic drug widely used to treat fever in infants, simethicone, a mixture of dimethicone and SiO_2_, is an inert antifoaming commonly used as a treatment to reduce bloating and abdominal discomfort associated with infantile colic^71–74^. Furthermore, the microbiota of each infant fecal sample was cultivated in IGSM without the addition of any drug to obtain the control sample. Samples were subjected to flow cytometry bacterial enumeration as well as to shallow shotgun sequencing generating a total of 772,908 reads (with an average of 51,527 reads per sample) which following chimera and quality filtering was reduced to a total of 637,447 reads and an average of 42,496 reads per sample (Supplementary Table 14). Specifically, flow cytometry readouts revealed no appreciable differences in total bacterial cell loads between the control and drug-treated samples (Supplementary Figure 3). At the same time, in depth insight into absolute abundance-based taxonomic profiles demonstrated that treatment with the abovementioned drugs did not induce any significant modification in the abundance of bacterial species above-identified as infant gut microbiota predominant taxa, except for *E. coli* whose abundance was shown to increase nearly ten times in I16 sample treated with simethicone (Supplementary Figure 3). Furthermore, although the abundance of certain accessory bacterial species underwent alterations when infant fecal samples are treated with drugs, no common trends were observed across the analyzed five fecal samples. These findings suggest that neither acetaminophen nor simethicone inhibit growth of the overall infant gut microbiota, and that neither drug seems, in general, to affect the taxonomic composition and abundance of infant gut microbial players. However, these analyses do not reveal if drug treatment did impact on infant gut microbiota metabolic functions. Therefore, a metabolomic analysis could make the analysis more comprehensive in order to fully understand the possible effects of drugs on the intestinal microbial community.

## Discussion

To investigate species-level taxonomic composition of the infant gut microbiota, a meta-analysis comprising 1669 publicly available metagenomes corresponding to fecal samples of healthy infants aged from few days to three years of life was performed. The re-analysis of these samples allowed us to identify the most abundant and prevalent bacterial species of the infant gut microbiota when samples are divided into 1-6 M, 6-12 M and 12-36 M age groups, leading to the identification of the infant “core” gut microbiota. At the same time, these data were further explored to assess the microbial taxonomic patterns of the infant gut microbial ecosystem, revealing the existence of distinct bacterial assemblies. Based on this data, infant fecal samples containing the here identified key bacterial species were cultivated on different growth media commonly used for the in vitro cultivation of the human gut microbiota. Subsequently, observed correlations between taxonomic profiles, bacterial total counts obtained for each 24 h cultivation, and the components of each culture medium allowed the identification of macro- and micro-nutrients necessary for growth of relevant bacterial components of the infant gut microbiota. Based on this correlation, an optimized culture medium was formulated, i.e., IGSM, as an optimized medium for the in vitro cultivation of the infant gut microbiota. Indeed, IGSM not only supports excellent growth performance after 24 h cultivation in terms of total bacterial counts, but it also sustains growth of most relevant species with respect to the inoculum. After IGSM validation, this culture medium was used to investigate the effect that drugs commonly used in pediatric age^71–74^ may have on infant gut microbiota taxonomic composition, i.e., acetaminophen and simethicone. Notably, such in vitro assay revealed that these drugs, unlike what has been demonstrated for other non-antibiotic drugs^75^, did not provoke any major change in the composition of the infant gut microbiota and seems to preserve the original bacterial composition of this very complex ecosystem.

## Methods

The STORMS (Strengthening The Organization and Reporting of Microbiome Studies) Checklist was adhered to for this study and is shown in Supplementary Table 15^76^.

### Ethical statement

The study protocol was approved by the Ethical Committee of the “Azienda Sanitaria Locale di Reggio Emilia–IRCCS” in Reggio Emilia, Italy as well as by the Ethical Committee of the University of Parma, Italy. Signed informed consents were obtained from the legally authorized representatives of each infant enrolled in this study.

### Database selection

One thousand and sixty-nine publicly available samples were obtained from 17 different studies aimed at characterizing the infant gut microbiota through the application of a shotgun metagenomics approach (Table 1 and Supplementary Table 1). To achieve high quality and coverage data, only shotgun metagenomics data sets based on Illumina sequencing technology were selected. Furthermore, only shotgun metagenomics samples involving fecal samples from healthy and full-term infants with an age ranging from one month to three years of life and not having undergone any drug, probiotic and prebiotic treatments were included in the study. No exclusion criteria based on mode of delivery, diet and geography were applied on the selection of the infant cohort.

### Shotgun metagenomic dataset analysis

To avoid biases caused by different bioinformatic analysis pipelines, the sequence read pools of each sample were filtered and analyzed by employing the METAnnotatorX2 software pipeline^26^. Taxonomic classification of up to 100,000 reads was obtained through megaBLAST^77^, employing a manually curated and pre-processed database of taxonomically validated RefSeq genomes retrieved from NCBI (https://www.ncbi.nlm.nih.gov/refseq/)^26^. Furthermore, microbial species were predicted using those Megablast hits (-evalue 1e-5, -qcov_hsp_perc 50) that unveiled a sequence identity above 94% in respect to the RefSeq (genome) database. Those reads that showed the same sequence identity against more than one bacterial species were discarded from the analysis to avoid species misclassification^26^.

Bray-Curtis dissimilarity index was used to estimate beta-diversity between different age groups. Dissimilarities were represented through a 3 (or 2)-dimensional Principal Coordinate Analysis (PCoA) through QIIME2^78,79^. Furthermore, infant gut community state types were predicted by means of a hierarchical clustering (HCL) analysis based on species-level bacterial composition of each enrolled sample and calculated through Origin 2021 software. The obtained data were represented through a cladogram.

### Subject recruitment, sample collection and gut microbiota culturing

Starting from the notion that the human gut microbiota is constantly evolving until the third year of life, when transition from an infant- to an adult-like gut microbiota typically occurs^3–5^, to cover the entire time window corresponding to the infant-like gut microbiota, five fecal samples were collected from infants aged three to 29 months (mean age of 14.2 months) (Supplementary Table 9). To be enrolled, infants had to be healthy and not having undergone treatment with any probiotics, prebiotics, or drugs during the three months prior to sample collection. Approximately three grams of fresh stool sample were collected from each infant immediately after defecation using a dedicated sterile tube provided of a sampling spoon and containing 15 ml of sterile PBS (phosphate-buffered saline, pH 6.5) pre-reduced with 0.1% (w/v) L-cysteine hydrochloride. After collection, stool samples were immediately shipped to the laboratory under anaerobic conditions and further processed as previously described^21^. Briefly, once in the laboratory, stool samples were transferred into an anaerobic workstation (2.99% H_2_, 17.01% CO_2_ and 80% N_2_) at 37 °C, where tubes were uncapped for a few seconds to allow gas exchange and removal of oxygen. Subsequently, stool samples were homogenized, gauze-filtered and immediately inoculated into 18 different culture media (Supplementary Table 10) at a final inoculum concentration of 2% (v/v). The pH of each culture medium was standardized to 6.8 to mimic the infant colon pH prior to autoclaving^80,81^, while vitamins and/or mineral solutions were sterilized by filtration using a 0.2 μm filter and added to culture media once cooled. Cultivation was carried out in 1 ml of each selected growth medium following the MiPro model^21^, i.e., involving 96-deep well plates covered with a silicone gel mat provided with a vent hole on each well, created by the use of a sterile syringe needle. During cultivation, plates were shaken at 500 rpm. For each infant fecal sample, two biological replicates were cultured for 24 h, while two replicates were obtained at 0 h (immediately after inoculation).

### Infant Gut Super Medium preparation and validation

The IGSM composition was defined based on total bacterial count and taxonomic analysis obtained from the cultivation of infant fecal samples on the 18 abovementioned culture media. Specifically, the Infant Gut Super Medium includes 3 g L^−1^ tryptone, 2 g L^−1^ casein, 4.5 g L^−1^ yeast extract, 2 g L^−1^ peptone, 1 g L^−1^ papaic digest of soyabean meal, 4.5 g L^−1^ digested serum, 0.75 g L^−1^ meat extract, 0.4 g L^−1^ liver extract, 0.2 g L^−1^ inulin, 0.2 g L^−1^ pectin, 0.2 g L^−1^ arabinogalactan, 1 g L^−1^ guar gum, 0.2 g L^−1^ xylan, 0.2 g L^−1^ lactose, 0.8 g L^−1^ L-cysteine hydrochloride, 3 g L^−1^ starch, 3 g L^−1^ mucin from porcine stomach type III, 2 g L^−1^ KCl, 2 g L^−1^ NaCl, 1 g L^−1^ NaHCO_3_, 0.05 g L^−1^ sodium cholate, 0.05 g L^−1^ sodium chenodeoxycholate, 0.0025 g L^−1^ hemin, 0.001 g L^−1^ FeSO_4_, 0.5 g L^−1^ MnSO_4_, 0.04 g L^−1^ KH_2_PO_4_, 0.04 g L^−1^ K_2_HPO_4_, 1 mL L^−1^ Tween 80. Final pH was adjusted to 6.8, while, after autoclaving and cooling, IGSM was supplemented with 0.2 μm filter-sterilized vitamin solution from medium 7 (1 mL L^−1^) and mineral solution from medium 3 (1 mL L^−1^) (Supplementary Table 10). To assess whether the optimized growth medium formulation was able to better maintain the taxonomic composition of the inoculum as compared to previously tested growth media, five additional fresh infant fecal samples were collected and cultivated in 96-deep well plates using IGSM as well as medium 1, 9, 11 and 15, following the above-described protocol. For this purpose, only fecal samples of healthy infants aged from one to six months and who had not undergone probiotic, prebiotic, or antibiotic treatments were collected (Supplementary Table 9). For each infant fecal sample, two biological replicates were cultured for 24 h, while two replicates were obtained at 0 h (immediately after inoculation).

### In vitro drug assay in IGSM

In vitro effects of active ingredients included in pharmacological formulations commonly used in early life on gut microbiota were assessed by culturing freshly collected infant fecal samples employed for IGSM validation assay, in the presence of two different compounds, i.e., acetaminophen and simethicone, following the MiPro model^21^. Cultivation was performed for 24 h and each compound was individually added to the IGSM with a final concentration of 2 μM, as previously described^82^. For each infant stool, a control sample, i.e., fecal inoculum in IGSM without any compounds, was obtained. Once collected, all samples were stored at -80 °C until they were processed for DNA extraction.

### DNA extraction and Illumina shallow shotgun sequencing

Each cultivation replicate was subjected to DNA extraction using the QIAmp DNA Stool Mini Kit following the manufacturer’s instructions (Qiagen, Germany). The extracted DNA was prepared using the Illumina Nextera XT DNA Library Preparation Kit and following the Illumina NexteraXT protocol. Briefly, DNA samples were enzymatically fragmented, barcoded, and purified employing magnetic beads. Subsequently, samples were quantified using fluorometric Qubit quantification system (Life Technologies, USA), loaded on a 2200 Tape Station Instrument (Agilent Technologies, USA) and normalized to 4 nM. A paired-end sequencing was performed using an Illumina MiSeq sequencer with MiSeq Reagent Kit v3 (Illumina Inc., San Diego, USA).

### Analysis of shallow shotgun metagenomic datasets

The obtained.fastq files were filtered to remove reads with a quality of <25 and sequences of human DNA, while reads with a length of >149 bp were retained. Quality-filtered data were used for further analysis with METAnnotatorX2 for taxonomic profile reconstruction, as previously described^26,83^.

### Evaluation of bacterial cell density by flow cytometry

For total cell counts, each culture replicate was 100,000 times diluted in physiological solution (PBS). Subsequently, 1 mL of the obtained bacterial cell suspension was stained with 1 μl of SYBR^®^Green I (ThermoFisher Scientific, USA) (1:100 dilution in dimethylsulfoxide; Sigma, Germany), vortex-mixed and incubated at 37 °C in the dark for at least 15 min before measurement. All count experiments were performed using an Attune NxT flow cytometry (ThermoFisher Scientific, Waltham, MA, USA) equipped with a blue laser set at 50 mW and tuned at an excitation wavelength of 488 nm. Multiparametric analyses were performed on both scattering signals, i.e., forward scatter (FSC) and side scatter (SSC), while SYBR Green I fluorescence was detected on the BL1 530/30 nm optical detector. Cell debris was excluded from acquisition analysis by setting a BL1 threshold. Furthermore, the gated fluorescence events were evaluated on the forward-sideways density plot to exclude remaining background events and to obtain an accurate microbial cell count, as previously described^50^. All data were statistically analyzed with the Attune NxT flow cytometry software.

### Statistical analyses

Origin 2021 (https://www.originlab.com/2021) was used to perform the Silhouette Width analysis as well as the hierarchical clustering of samples using bacterial composition at species level and Pearson correlation as distance metric. QIIME2 was exploited for both PCoA, and ANOSIM calculation^78,79^. SPSS software (www.ibm.com/software/it/analytics/spss) was employed to compute statistical analyses, including ANOVA and the LSD *post hoc* analysis was calculated for multiple comparison, while covariance analysis was calculated through Pearson coefficient correlation. Furthermore, the R studios software (version 4.1.2) was employed to perform Bray-Curtis dissimilarity matrix and Prediction Strength analysis through the use of various packages, including data.table (version 1.14.2) to load the initial data, fpc (version 2.2-9) to perform the Prediction strength analysis, devtools (version 2.4.3) (https://github.com/r-lib/devtools), vegan (version 2.5-7) to calculate Bray-Curtis matrices, dplyr (version 1.0.8) to ensure compatibility in data processing and command writing (https://dplyr.tidyverse.org, https://github.com/tidyverse/dplyr), pbapply (version 1.5-0) to add progress bar to “*apply” functions in R (https://github.com/psolymos/pbapply), and cluster (version 2.1.2) required for cluster analysis. Furthermore, Prediction Strength was calculated by cross-validation on all samples pooled together.

### Reporting summary

Further information on research design is available in the Nature Research Reporting Summary linked to this article.

## Supplementary information

Supplementary Material

Supplementary Tables

Reporting Summary

## Data Availability

Raw sequences of shallow shotgun sequences are accessible through Sequence Read Archive (SRA) under BioProject accession number PRJNA782810.

## Code availability

No informatic code were used for this study.
