# Supplementary material for: Exploring species-level infant gut bacterial biodiversity by meta-analysis and formulation of an optimized cultivation medium
Source: NPJ Biofilms Microbiomes. 2022 Oct 31;8:88. doi: 10.1038/s41522-022-00349-1 (PMC9622858; doi:10.1038/s41522-022-00349-1)
Supplement: Supplementary file 1 — Supplementary Material [file 41522_2022_349_MOESM1_ESM.pdf]

Exploring species-level infant gut bacterial biodiversity by meta-analysis and formulation of an optimized cultivation medium

Running title: growth medium for infant gut microbiota cultivation

Key words: meta-analysis, infants, gut microbiota, *in vitro* cultivation, growth medium, drugs and microbiota

Giulia Alessandri[1], Federico Fontana[2], Leonardo Mancabelli[1], Gabriele Andrea Lugli[1], Chiara Tarracchini[1], Chiara Argentini[1], Giulia Longhi[2], Alice Viappiani[2], Christian Milani[1,3], Francesca Turroni[1,3], Douwe van Sinderen[4] and Marco Ventura[1,3*]

[1]Laboratory of Probiogenomics, Department of Chemistry, Life Sciences, and Environmental Sustainability, University of Parma, Parma, Italy; [2]GenProbio srl, Parma, Italy; [3]Microbiome Research Hub, University of Parma, Parma, Italy; [4]APC Microbiome Institute and School of Microbiology, Bioscience Institute, National University of Ireland, Cork, Ireland

[*]Corresponding author

Mailing address for Marco Ventura, Laboratory of Probiogenomics, Department of Chemistry, Life Sciences, and Environmental Sustainability, University of Parma, Parco Area delle Scienze 11a, 43124 Parma, Italy. Phone: ++39-521-905666. Fax: ++39-521-905604. E-mail: marco.ventura@unipr.it

Supplementary text

Selection of public datasets. To formulate an *ad hoc* culture medium suitable for *in vitro* cultivation of the infant gut microbiota, a meta-analysis aimed at assessing the taxonomic composition of the infant intestinal microbiota based on all publicly available shotgun metagenomics datasets was performed. In this context, an in-depth literature search was performed to select shotgun metagenomics datasets based on Illumina sequencing technology corresponding to fecal samples from healthy, full-term infants. Specifically, exclusively shotgun metagenomics-based datasets were selected in order to i) obtain high quality and coverage data, ii) eliminate biases associated with 16S rRNA gene-based microbial profiling, principally due to the lack of agreement among the scientific community about which primer pairs are most appropriate for the amplification of the 16S rRNA gene or which region(s) of the latter gene is(are) to be targeted to obtain the best sequencing efficiency and, iii) provide a high resolution characterization of the infant gut microbiota with high accuracy down to the species-level[1-4]. Furthermore, to cover the entire life period characterized by an infant-like gut microbiota, publicly available datasets corresponding to fecal samples of infants aged between few days and three years were considered. Indeed, weaning and concomitant transition from a milk-based to a solid and more varied diet prompts a profound change in the taxonomic composition of the infant gut microbiota with an overall increase in bacterial biodiversity, and it is around the third year of life that the transition from an infant- to an adult-like gut microbiota is thought to occur[5-9]. Based on these parameters, 2411 publicly available datasets were selected from 17 cohorts covering different geographical areas. However, in case of longitudinally studies, only one sample per infant was considered to avoid redundant samples, while in case of studies involving the administration of drugs, prebiotics or probiotics, only infant fecal samples belonging to the control group were taken into account.

Meta-analysis of the infant gut bacterial community and reconstruction of the species-level infant "core" gut microbiota. Species richness analysis revealed a progressive and statistically significant

increment in the average number of species across groups that positively correlates with increasing age (ANOVA post-hoc p-value < 0.01) (Figure 1). Specifically, while the 1-6M group showed an average of 15 bacterial species per sample, an average of 19 and 24 bacterial taxa was observed for the 6-12M and 12-36M group, respectively (Figure 1). This finding not only corroborates the already well accepted notion that the infant gut microbiota gradually diversifies from birth to the third year of life, but it also emphasizes the relevant role of weaning in shaping the human intestinal ecosystem and its contribution in increasing intestinal biodiversity[8,10-13]. Similarly, a PCoA representation-based beta-diversity analysis highlighted clear compositional differences between samples belonging to 1-6M and 12-36M groups, while 6-12M samples did not create a separate group. Rather, they seemed to cluster with samples of either of the other two groups (Figure 1), thus strengthening the pivotal role of weaning in influencing the taxonomic composition of the infant gut microbiota. Indeed, while the separation of samples belonging to the 1-6M and 12-36M may be attributable to the different diets followed by infants during these two age groups, i.e. an exclusively milk-based diet for the first group and a solid and varied diet for the second group, from the sixth month to the first year of life, infants go through a transition phase (weaning) during which they are supposed to follow a 'hybrid' diet characterized by milk assumption and introduction of complementary feeding resulting in a bacterial composition oscillating between that of the other two groups[14-18].

Furthermore, to evaluate how and to what extent diet and/or geographic location may impact on the infant intestinal microbial composition, the "core" gut microbiota was further assessed subdividing samples according to the geographic origin per each considered age group. Specifically, to provide more robustness to the analysis, only groups with at least 10 samples based on their geographical origin were considered. Interestingly, all subgroups of the 1-6M group were characterized by a simultaneous high relative abundance and prevalence of the four main species identified as "core" gut microbiota of infants aged between one and six months, i.e., *B. bifidum*, *B. breve*, *B. longum*, and *E. coli* (Supplementary Table

4). This finding suggests that the latter species can be considered as infant gut microbial players typical of the very first months of life regardless of geographic origin. However, the subdivision of 1-6M samples according to their geographical origins revealed that four additional species, i.e., *Streptococcus salivarius*, *Prevotella copri*, *Klebsiella pneumoniae*, and *Blautia wexelerae*, not previously identified, displayed a prevalence >40% in four different 1-6M subgroups, suggesting that these species are typical of the intestinal ecosystem of 1-6M infants of a specific nationality (Supplementary Table 4). As observed for 1-6M infants, also fecal samples of the 6-12M group showed a high prevalence of the bacterial species identified as "core" gut microbiota in weaning infants regardless of geographical origin, except for fecal samples from South African or Malawian infants. Indeed, the latter showed a partial variation of the "core" gut microbiota with a reduced prevalence or total absence of *B. wexelerae* as well as of most of the bacterial species previously identified as accessory gut microbiota (Supplementary Table 4). In this context, while the geographical location does not appear to affect the composition of the core gut microbiota when diet is exclusively based on milk, different geographical origin, and therefore different dietary habits, may modulate the intestinal microbial ecosystem when the passage from a liquid to a solid diet occurs[19]. Finally, the two geographic origin-based subgroups of the 12-36M group were characterized by the presence of all bacterial species identified as "core" gut microbiota of the 12-36M infants with a reduced prevalence of species belonging to the genus *Bacteroides* for fecal samples of New Zealand infants (Supplementary Table 4). This finding indicates that, for this age group, diet or geographical origin seems to play a marginal role in the modulation of the prevalent intestinal bacterial species. However, a higher number of groups by geographic origin would help to better understand the impact of diet and nationality on the intestinal ecosystem of 12-36M infants.

Altogether, these data demonstrate that diet and geographical origin may play a role in the modulation of the infant gut microbiota, but they do not drastically affect the prevalent species identified as "core" gut microbiota of each age group.

Identification of species-level Infant Gut Community State Types (sIGCSTs). To validate the identified sIGCSTs and sub-sIGCSTs, a two-way frequency table was employed to assess if the geographic origin or the cohort to which each fecal sample belongs to could represent a bias in the subdivision of samples into sIGCSTs and ssIGCSTs. By considering the sIGCSTs, certain observed frequencies of samples within a cluster differ from the calculated expected frequency based on both geographic origin and study cohort (Supplementary Table 7). Indeed, especially sIGCST2 and sIGCST4 showed some frequencies related to samples from Finland/Estonia/Russia and Malawi that were two-fold higher or lower than predicted. Thus suggesting, as expected, that geographic origin and study cohort may have a role in influencing the taxonomic composition and metagenomic data related to this microbial ecosystem, respectively [20-22]. However, since no cluster resulted to be exclusively represented by samples from a single nation or cohort, the identified clusters can be considered as real discrete compositional patterns.

Furthermore, the same analysis was conducted to verify and validate the identified ssIGCSTs. As above observed for sIGCSTs, also for the sub-clusters, the two-way frequency analysis highlighted that various observed frequencies varied from those expected for both the considered factors, thus reinforcing the role of geographic origin and study cohort in influencing the subdivision of samples into clusters. However, also in this case, no clusters included samples exclusively related to a single nation or bioproject, with the exception of cluster 7 and 10 which were represented by more than 90% of samples from a specific geographical origin or study cohort (Supplementary Table 7). Thus, clusters 7 and 10 were excluded from the identified ssIGCSTs, while all the other clusters were considered as putative compositional motifs of the infant gut microbiota.

References

1 Milani, C. *et al.* Assessing the fecal microbiota: an optimized ion torrent 16S rRNA gene-based analysis protocol. *PLoS One* **8**, e68739, doi:10.1371/journal.pone.0068739 (2013).

2 Mancabelli, L. *et al.* The Impact of Primer Design on Amplicon-Based Metagenomic Profiling Accuracy: Detailed Insights into Bifidobacterial Community Structure. *Microorganisms* **8**, doi:10.3390/microorganisms8010131 (2020).

3 Boers, S. A., Jansen, R. & Hays, J. P. Understanding and overcoming the pitfalls and biases of next-generation sequencing (NGS) methods for use in the routine clinical microbiological diagnostic laboratory. *Eur J Clin Microbiol Infect Dis* **38**, 1059-1070, doi:10.1007/s10096-019-03520-3 (2019).

4 Alessandri, G. *et al.* Catching a glimpse of the bacterial gut community of companion animals: a canine and feline perspective. *Microb Biotechnol* **13**, 1708-1732, doi:10.1111/1751-7915.13656 (2020).

5 Mueller, N. T., Bakacs, E., Combellick, J., Grigoryan, Z. & Dominguez-Bello, M. G. The infant microbiome development: mom matters. *Trends Mol Med* **21**, 109-117, doi:10.1016/j.molmed.2014.12.002 (2015).

6 Sandyk, R. Zinc deficiency in attention-deficit hyperactivity disorder. *Int J Neurosci* **52**, 239-241, doi:10.3109/00207459009000526 (1990).

7 Derrien, M., Alvarez, A. S. & de Vos, W. M. The Gut Microbiota in the First Decade of Life. *Trends Microbiol* **27**, 997-1010, doi:10.1016/j.tim.2019.08.001 (2019).

8 Mancabelli, L. *et al.* Multi-population cohort meta-analysis of human intestinal microbiota in early life reveals the existence of infant community state types (ICSTs). *Comput Struct Biotechnol J* **18**, 2480-2493, doi:10.1016/j.csbj.2020.08.028 (2020).

9 Alessandri, G., Ossiprandi, M. C., MacSharry, J., van Sinderen, D. & Ventura, M. Bifidobacterial Dialogue With Its Human Host and Consequent Modulation of the Immune System. *Front Immunol* **10**, 2348, doi:10.3389/fimmu.2019.02348 (2019).

10 Fallani, M. *et al.* Determinants of the human infant intestinal microbiota after the introduction of first complementary foods in infant samples from five European centres. *Microbiology (Reading)* **157**, 1385-1392, doi:10.1099/mic.0.042143-0 (2011).

11 Koenig, J. E. *et al.* Succession of microbial consortia in the developing infant gut microbiome. *Proc Natl Acad Sci U S A* **108 Suppl 1**, 4578-4585, doi:10.1073/pnas.1000081107 (2011).

12 Stanislawski, M. A. *et al.* Gut Microbiota in the First 2 Years of Life and the Association with Body Mass Index at Age 12 in a Norwegian Birth Cohort. *mBio* **9**, doi:10.1128/mBio.01751-18 (2018).

13 Ihekweazu, F. D. & Versalovic, J. Development of the Pediatric Gut Microbiome: Impact on Health and Disease. *Am J Med Sci* **356**, 413-423, doi:10.1016/j.amjms.2018.08.005 (2018).

14 Bergstrom, A. *et al.* Establishment of intestinal microbiota during early life: a longitudinal, explorative study of a large cohort of Danish infants. *Appl Environ Microbiol* **80**, 2889-2900, doi:10.1128/AEM.00342-14 (2014).

15 Laursen, M. F., Bahl, M. I., Michaelsen, K. F. & Licht, T. R. First Foods and Gut Microbes. *Front Microbiol* **8**, 356, doi:10.3389/fmicb.2017.00356 (2017).

16 Backhed, F. *et al.* Dynamics and Stabilization of the Human Gut Microbiome during the First Year of Life. *Cell Host Microbe* **17**, 690-703, doi:10.1016/j.chom.2015.04.004 (2015).

17 Tanaka, M. & Nakayama, J. Development of the gut microbiota in infancy and its impact on health in later life. *Allergol Int* **66**, 515-522, doi:10.1016/j.alit.2017.07.010 (2017).

18 Efsa Panel on Nutrition, N. F. *et al.* Appropriate age range for introduction of complementary feeding into an infant's diet. *EFSA J* **17**, e05780, doi:10.2903/j.efsa.2019.5780 (2019).

19 De Filippo, C. *et al.* Impact of diet in shaping gut microbiota revealed by a comparative study in children from Europe and rural Africa. *Proc Natl Acad Sci U S A* **107**, 14691-14696, doi:10.1073/pnas.1005963107 (2010).

20 De Filippo, C. *et al.* Diet, Environments, and Gut Microbiota. A Preliminary Investigation in Children Living in Rural and Urban Burkina Faso and Italy. *Front Microbiol* **8**, 1979, doi:10.3389/fmicb.2017.01979 (2017).

21 Tourlousse, D. M. *et al.* Characterization and Demonstration of Mock Communities as Control Reagents for Accurate Human Microbiome Community Measurements. *Microbiol Spectr* **10**, e0191521, doi:10.1128/spectrum.01915-21 (2022).

22 Wagner Mackenzie, B., Waite, D. W. & Taylor, M. W. Evaluating variation in human gut microbiota profiles due to DNA extraction method and inter-subject differences. *Front Microbiol* **6**, 130, doi:10.3389/fmicb.2015.00130 (2015).

a)

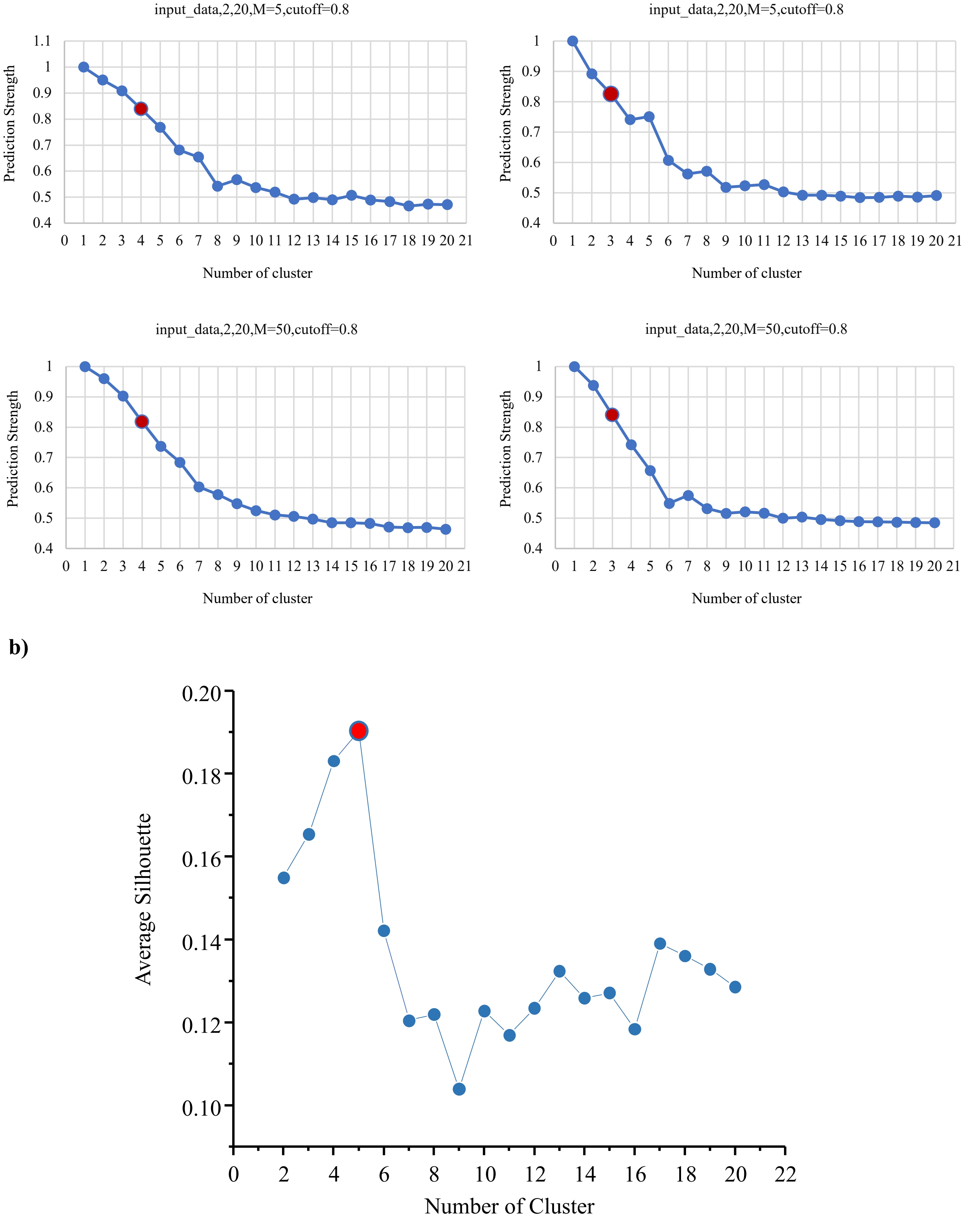

Supplementary Figure 1: Unsupervised cluster validation analyses. Panel a reports Prediction Strength analyses calculated with kmeans clustering method (panels on the left) and with clara/pam clustering method (panels on the right) both with 5 (top panels) and 50 (bottom panels) resampled data sets. Panel b displays the Silhouette Width analysis.

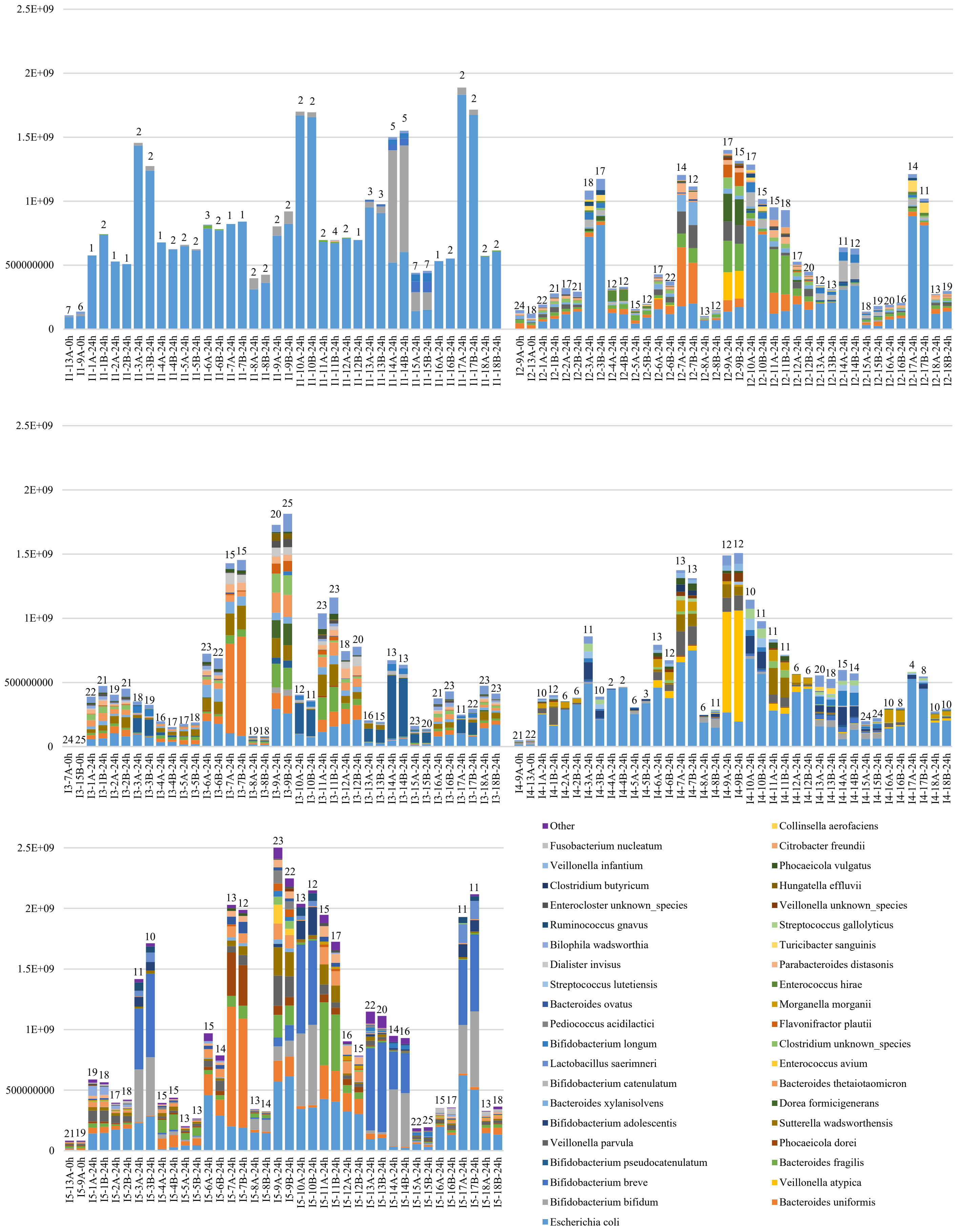

Supplementary Figure 2: *In vitro* growth performances of infant fecal samples in different culture media. Each bar plot-based graph is related to one of the five infant fecal samples cultivated in 18 different culture media. The y-axis reports flow-cytometry based bacterial cell enumeration, while bar plot colors correspond to a specific bacterial species. Numbers reported on bar plots refer to the number of species detected in each replicate considering only those species with a relative abundance >0.5%.

a)

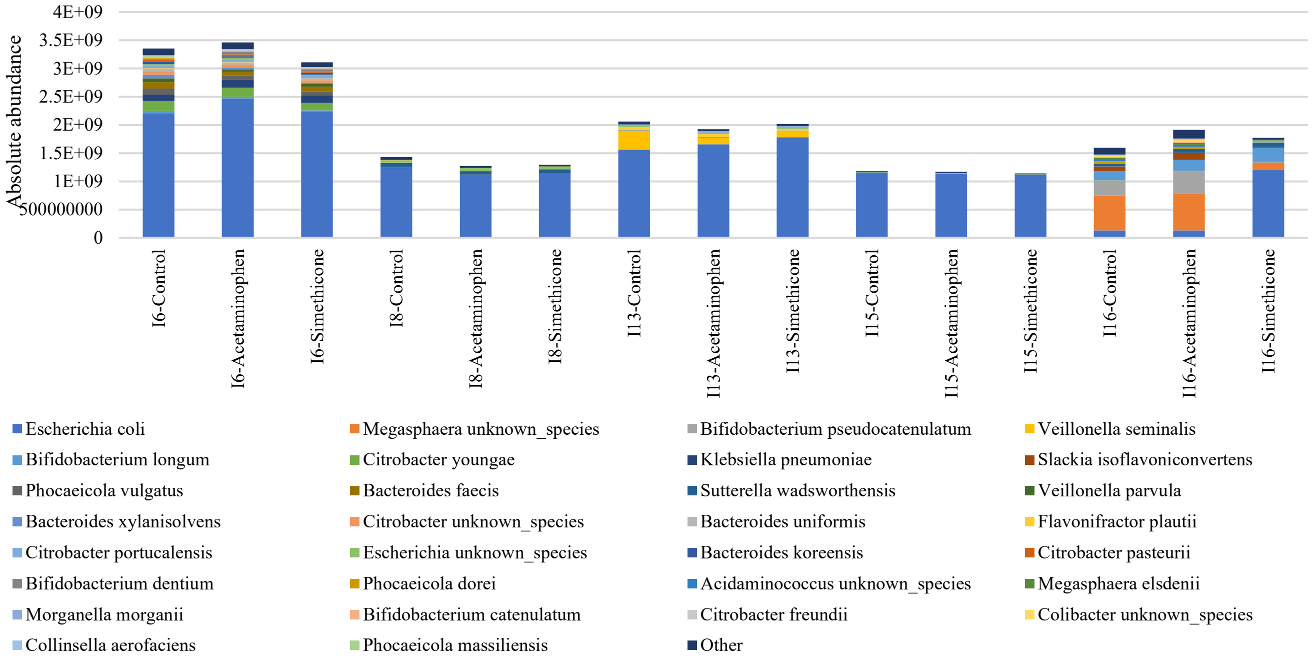

b)

Reads Taxonomy	I6-C	I6-A	C/A	I6-S	S/A	I8-C	I8-A	C/A	I8-S	S/A	I13-C	I13-A	C/A	I13-S	S/A	I15-C	I15-A	C/A	I15-S	S/A	I16-C	I16-A	C/A	I16-S	S/A
Bacteroides faecis	1.1E+08	6.6E+07	1.64	8.1E+07	1.35	0.0E+00	0.0E+00		0.0E+00		0.0E+00	0.0E+00		0.0E+00		0.0E+00	0.0E+00		0.0E+00		0.0E+00	0.0E+00		0.0E+00	
Bacteroides koreensis	4.2E+07	2.4E+07	1.72	3.5E+07	1.20	0.0E+00	0.0E+00		0.0E+00		0.0E+00	0.0E+00		0.0E+00		0.0E+00	0.0E+00		0.0E+00		0.0E+00	0.0E+00		0.0E+00	
Bifidobacterium longum	4.1E+07	3.0E+07	1.35	2.3E+07	1.78	3.0E+06	0.0E+00		0.0E+00		1.2E+07	8.6E+06	1.42	8.6E+06	1.43	4.2E+06	7.4E+06	0.57	4.6E+06	0.91	1.7E+08	1.9E+08	0.88	2.5E+08	0.66
Citrobacter amalonaticus	1.7E+07	1.7E+07	1.00	1.1E+07	1.54	0.0E+00	0.0E+00		0.0E+00		0.0E+00	0.0E+00		0.0E+00		0.0E+00	0.0E+00		0.0E+00		0.0E+00	0.0E+00		0.0E+00	
Citrobacter braakii	1.0E+07	9.6E+06	1.07	5.6E+06	1.83	0.0E+00	0.0E+00		0.0E+00		0.0E+00	0.0E+00		0.0E+00		0.0E+00	0.0E+00		0.0E+00		0.0E+00	0.0E+00		0.0E+00	
Citrobacter freundii	2.6E+07	2.7E+07	0.94	2.3E+07	1.14	0.0E+00	0.0E+00		0.0E+00		0.0E+00	0.0E+00		0.0E+00		0.0E+00	0.0E+00		0.0E+00		0.0E+00	0.0E+00		0.0E+00	
Citrobacter pasteurii	4.0E+07	3.8E+07	1.04	3.3E+07	1.23	0.0E+00	0.0E+00		0.0E+00		0.0E+00	0.0E+00		0.0E+00		0.0E+00	0.0E+00		0.0E+00		0.0E+00	0.0E+00		0.0E+00	
Citrobacter portucalensis	4.7E+07	5.5E+07	0.85	3.7E+07	1.27	0.0E+00	0.0E+00		0.0E+00		0.0E+00	0.0E+00		0.0E+00		0.0E+00	0.0E+00		0.0E+00		0.0E+00	0.0E+00		0.0E+00	
Citrobacter youngae	1.8E+08	1.6E+08	1.07	1.3E+08	1.38	0.0E+00	0.0E+00		0.0E+00		0.0E+00	0.0E+00		0.0E+00		0.0E+00	0.0E+00		0.0E+00		0.0E+00	0.0E+00		0.0E+00	
Klebsiella pneumoniae	1.1E+08	1.5E+08	0.74	1.3E+08	0.87	0.0E+00	0.0E+00		0.0E+00		0.0E+00	0.0E+00		0.0E+00		0.0E+00	0.0E+00		0.0E+00		0.0E+00	0.0E+00		0.0E+00	
Phocaeicola vulgatus	1.1E+08	6.5E+07	1.71	7.8E+07	1.43	6.4E+06	4.4E+06	1.45	4.7E+06	1.35	0.0E+00	0.0E+00		0.0E+00		0.0E+00	0.0E+00		0.0E+00		4.5E+06	4.1E+06	1.08	0.0E+00	
Bifidobacterium pseudocatenulatum	0.0E+00	0.0E+00		0.0E+00		3.5E+06	0.0E+00		0.0E+00		0.0E+00	0.0E+00		0.0E+00		0.0E+00	0.0E+00		0.0E+00		2.6E+08	4.0E+08	0.65	2.1E+07	12.03
Megasphaera elsdenii	0.0E+00	0.0E+00		0.0E+00		0.0E+00	0.0E+00		0.0E+00		0.0E+00	0.0E+00		0.0E+00		0.0E+00	0.0E+00		0.0E+00		2.9E+07	3.4E+07	0.86	6.9E+06	4.23
Phocaeicola dorei	2.1E+07	1.1E+07	1.85	1.2E+07	1.74	2.0E+06	1.5E+06	1.28	1.4E+06	1.41	0.0E+00	0.0E+00		0.0E+00		0.0E+00	0.0E+00		0.0E+00		3.7E+07	3.5E+07	1.07	9.2E+06	4.06
Slackia isoflavoniconvertens	0.0E+00	0.0E+00		0.0E+00		0.0E+00	0.0E+00		0.0E+00		0.0E+00	0.0E+00		0.0E+00		0.0E+00	0.0E+00		0.0E+00		7.9E+07	1.2E+08	0.63	1.4E+07	5.78
Escherichia coli	2.2E+09	2.5E+09	0.90	2.2E+09	0.99	1.2E+09	1.1E+09	1.10	1.1E+09	1.09	1.6E+09	1.7E+09	0.94	1.8E+09	0.88	1.2E+09	1.1E+09	1.02	1.1E+09	1.04	1.3E+08	1.3E+08	1.01	1.2E+09	0.11
Veillonella seminalis	0.0E+00	0.0E+00		0.0E+00		0.0E+00	0.0E+00		0.0E+00		3.4E+08	1.2E+08	2.73	1.1E+08	2.97	0.0E+00	0.0E+00		0.0E+00		5.3E+06	7.5E+06	0.70	0.0E+00	
Flavonifractor plautii	8.3E+06	6.1E+06	1.36	4.2E+06	1.99	0.0E+00	0.0E+00		0.0E+00		5.5E+07	5.2E+07	1.05	2.5E+07	2.18	0.0E+00	0.0E+00		0.0E+00		0.0E+00	0.0E+00		0.0E+00	
Sutterella wadsworthensis	0.0E+00	0.0E+00		0.0E+00		7.7E+07	5.3E+07	1.45	7.0E+07	1.10	0.0E+00	0.0E+00		0.0E+00		0.0E+00	0.0E+00		0.0E+00		4.9E+07	6.3E+07	0.77	7.7E+07	0.63
Veillonella parvula	6.7E+07	4.2E+07	1.59	5.0E+07	1.35	0.0E+00	0.0E+00		0.0E+00		0.0E+00	0.0E+00		0.0E+00		0.0E+00	0.0E+00		0.0E+00		0.0E+00	0.0E+00		0.0E+00	
Morganella morganii	0.0E+00	0.0E+00		0.0E+00		0.0E+00	0.0E+00		0.0E+00		2.4E+07	3.1E+07	0.78	2.9E+07	0.84	0.0E+00	0.0E+00		0.0E+00		0.0E+00	0.0E+00		0.0E+00	
Bacteroides xylanisolvens	6.3E+07	3.1E+07	2.03	2.2E+07	2.89	0.0E+00	0.0E+00		0.0E+00		0.0E+00	0.0E+00		0.0E+00		0.0E+00	0.0E+00		0.0E+00		3.4E+06	2.8E+06	1.25	3.8E+06	0.92
Bifidobacterium dentium	7.9E+06	4.0E+07	0.20	2.8E+07	0.28	0.0E+00	0.0E+00		0.0E+00		0.0E+00	0.0E+00		0.0E+00		0.0E+00	0.0E+00		0.0E+00		0.0E+00	0.0E+00		0.0E+00	
Parabacteroides distasonis	8.3E+06	4.3E+06	1.95	3.6E+06	2.32	0.0E+00	0.0E+00		0.0E+00		0.0E+00	0.0E+00		0.0E+00		0.0E+00	0.0E+00		0.0E+00		4.8E+06	8.7E+06	0.55	0.0E+00	
Phocaeicola massiliensis	2.1E+07	1.2E+07	1.83	1.0E+07	2.10	0.0E+00	0.0E+00		0.0E+00		0.0E+00	0.0E+00		0.0E+00		0.0E+00	0.0E+00		0.0E+00		0.0E+00	0.0E+00		0.0E+00	
Escherichia albertii	4.2E+06	5.1E+06	0.82	5.7E+06	0.73	1.9E+07	7.7E+06	2.49	6.9E+06	2.77	6.9E+06	6.4E+06	1.08	6.6E+06	1.05	3.4E+06	2.4E+06	1.38	3.2E+06	1.06	0.0E+00	0.0E+00		4.2E+06	
Clostridium neonatale	0.0E+00	0.0E+00		0.0E+00		0.0E+00	0.0E+00		0.0E+00		9.0E+06	3.8E+06	2.37	5.8E+06	1.55	0.0E+00	0.0E+00		0.0E+00		0.0E+00	0.0E+00		0.0E+00	
Bacteroides uniformis	5.6E+07	3.6E+07	1.56	3.4E+07	1.61	0.0E+00	0.0E+00		0.0E+00		0.0E+00	0.0E+00		0.0E+00		0.0E+00	0.0E+00		0.0E+00		0.0E+00	1.2E+06		0.0E+00	
Other	1.6E+08	1.6E+08		1.2E+08		7.9E+07	8.0E+07		7.3E+07		5.6E+07	4.6E+07		4.5E+07		1.9E+07	3.0E+07		2.1E+07		8.2E+08	9.1E+08		1.8E+08	

Supplementary Figure 3: *In vitro* growth performances of infant fecal samples in different culture media. Each bar plot-based graph is related to one of the five infant fecal samples cultivated in 18 different culture media. The y-axis reports flow-cytometry based bacterial cell enumeration, while bar plot colors correspond to a specific bacterial species. Numbers reported on bar plots refer to the number of species detected in each replicate considering only those species with a relative abundance >0.5%.
