# Supplementary material for: Exploring species-level infant gut bacterial biodiversity by meta-analysis and formulation of an optimized cultivation medium
Source: NPJ Biofilms Microbiomes. 2022 Oct 31;8:88. doi: 10.1038/s41522-022-00349-1 (PMC9622858; doi:10.1038/s41522-022-00349-1)
Supplement: Supplementary file 3 — Reporting Summary [file 41522_2022_349_MOESM3_ESM.pdf]

nature portfolio

Corresponding author(s): Prof. Marco Ventura
Last updated by author(s): Jun 22, 2022

Reporting Summary

Nature Portfolio wishes to improve the reproducibility of the work that we publish. This form provides structure for consistency and transparency in reporting. For further information on Nature Portfolio policies, see our Editorial Policies and the Editorial Policy Checklist.

Statistics

For all statistical analyses, confirm that the following items are present in the figure legend, table legend, main text, or Methods section.

n/a	Confirmed	
☐	☒	The exact sample size (*n*) for each experimental group/condition, given as a discrete number and unit of measurement
☐	☒	A statement on whether measurements were taken from distinct samples or whether the same sample was measured repeatedly
☐	☒	The statistical test(s) used AND whether they are one- or two-sided<br>*Only common tests should be described solely by name; describe more complex techniques in the Methods section.*
☐	☒	A description of all covariates tested
☐	☒	A description of any assumptions or corrections, such as tests of normality and adjustment for multiple comparisons
☐	☒	A full description of the statistical parameters including central tendency (e.g. means) or other basic estimates (e.g. regression coefficient) AND variation (e.g. standard deviation) or associated estimates of uncertainty (e.g. confidence intervals)
☐	☒	For null hypothesis testing, the test statistic (e.g. *F*, *t*, *r*) with confidence intervals, effect sizes, degrees of freedom and *P* value noted<br>*Give P values as exact values whenever suitable.*
☒	☐	For Bayesian analysis, information on the choice of priors and Markov chain Monte Carlo settings
☐	☒	For hierarchical and complex designs, identification of the appropriate level for tests and full reporting of outcomes
☒	☐	Estimates of effect sizes (e.g. Cohen's *d*, Pearson's *r*), indicating how they were calculated

Our web collection on statistics for biologists contains articles on many of the points above.

Software and code

Policy information about availability of computer code

Data collection	No custom algorithms or software were used.
Data analysis	To avoid biases caused by different bioinformatic analysis pipelines, the sequence read pools of each sample were filtered and analyzed by employing the novel METAnnotatorX2 software pipeline. Taxonomic classification of up to 100,000 reads was obtained through megaBLAST, employing a manually curated and pre-processed database of taxonomically validated RefSeq genomes retrieved from NCBI (https://www.ncbi.nlm.nih.gov/refseq/). Furthermore, microbial species were predicted using those Megablast hits (-evalue 1e-5, -qcov_hsp_perc 50) that unveiled a sequence identity above 94% in respect to the RefSeq (genome) database. Those reads that showed the same sequence identity against more than one bacterial species were discarded from the analysis to avoid species misclassification. Bray-Curtis dissimilarity index was used to estimate beta-diversity between different age groups. Dissimilarities were represented through a 3 (or 2)-dimensional Principal Coordinate Analysis (PCoA) through QIIME2. Furthermore, infant gut community state types were predicted by means of a hierarchical clustering (HCL) analysis based on species-level bacterial composition of each enrolled sample and calculated through Origin 2021 software.

For manuscripts utilizing custom algorithms or software that are central to the research but not yet described in published literature, software must be made available to editors and reviewers. We strongly encourage code deposition in a community repository (e.g. GitHub). See the Nature Portfolio guidelines for submitting code & software for further information.

Data

Policy information about availability of data

All manuscripts must include a data availability statement. This statement should provide the following information, where applicable:

- Accession codes, unique identifiers, or web links for publicly available datasets
- A description of any restrictions on data availability
- For clinical datasets or third party data, please ensure that the statement adheres to our policy

Raw sequences of shallow shotgun sequences are accessible through Sequence Read Archive (SRA) under BioProject accession number PRJNA782810.

Human research participants

Policy information about studies involving human research participants and Sex and Gender in Research.

Reporting on sex and gender	the sex of infants from which fecal samples were collected was annotated in the supplementary tables. however, no analysis was performed on the basis of the sex of the involved subjects.
Population characteristics	For public metagenomic datasets, all relevant population characteristics (e.g., age and geographical origin) were retrieved from the publicly available associated study. For the 10 infants whose fecal samples were used for culture-based approaches, information about sex, age and geographical origin were collected directly from parents.
Recruitment	Five fecal samples were collected from infants aged three to 29 months (mean age of 14.2 months). To be enrolled, infants had to be healthy and not having undergone treatment with any probiotics, prebiotics, or drugs during the three months prior to sample collection.
Ethics oversight	The study protocol was approved by the Ethical Committee of the "Azienda Sanitaria Locale di Reggio Emilia – IRCCS" in Reggio Emilia, Italy as well as by the Ethical Committee of the University of Parma, Italy

Note that full information on the approval of the study protocol must also be provided in the manuscript.

Field-specific reporting

Please select the one below that is the best fit for your research. If you are not sure, read the appropriate sections before making your selection.

☒ Life sciences ☐ Behavioural & social sciences ☐ Ecological, evolutionary & environmental sciences

For a reference copy of the document with all sections, see nature.com/documents/nr-reporting-summary-flat.pdf

Life sciences study design

All studies must disclose on these points even when the disclosure is negative.

Sample size	For meta-analysis - One thousand and sixty-nine publicly available samples were obtained from 17 different studies aimed at characterizing the infant gut microbiota through the application of a shotgun metagenomics approach. To achieve high quality and coverage data, only shotgun metagenomics data sets based on Illumina sequencing technology were selected.<br>For microbiota culturing - Five fecal samples were collected from infants aged three to 29 months comprising the identified ssIGCST-dominant species. In addition other five fecal samples belonging to infants aged from one to six months and possessing the 1-6M group ssIGCST-characterizing bacterial species were retrieved.
Data exclusions	Only shotgun metagenomics samples involving fecal samples from healthy and full-term infants with an age ranging from one month to three years of life and not having undergone any drug, probiotic and prebiotic treatments were included in the study. No exclusion criteria based on mode of delivery, diet and geography were applied on the selection of the infant cohort.
Replication	All analysis were succesfully replicated at least twice. In addition, all growth experiments were performed in duplicate.
Randomization	For meta-analysis, based on the assumption that the infant gut microbiota is highly variable and dynamic during the first three years of life, the selected samples were divided into three different age-based groups, i.e., group 1-6M (from 30 days to 6 months of life), group 6-12M (between 6 months and one year of life), and group 12-36M (from one to three years of age).
Blinding	Blinding was not applicable.

Reporting for specific materials, systems and methods

We require information from authors about some types of materials, experimental systems and methods used in many studies. Here, indicate whether each material, system or method listed is relevant to your study. If you are not sure if a list item applies to your research, read the appropriate section before selecting a response.

Materials & experimental systems

n/a	Involved in the study	
☒	☐	Antibodies
☒	☐	Eukaryotic cell lines
☒	☐	Palaeontology and archaeology
☒	☐	Animals and other organisms
☒	☐	Clinical data
☒	☐	Dual use research of concern

Methods

n/a	Involved in the study	
☒	☐	ChIP-seq
☐	☒	Flow cytometry
☒	☐	MRI-based neuroimaging

Flow Cytometry

Plots

Confirm that:

☐ The axis labels state the marker and fluorochrome used (e.g. CD4-FITC).

☐ The axis scales are clearly visible. Include numbers along axes only for bottom left plot of group (a 'group' is an analysis of identical markers).

☐ All plots are contour plots with outliers or pseudocolor plots.

☒ A numerical value for number of cells or percentage (with statistics) is provided.

Methodology

Sample preparation	Each culture replicate was 100,000 times diluted in physiological solution (PBS). Subsequently, 1 mL of the obtained bacterial cell suspension was stained with 1 µl of SYBR®Green I (ThermoFisher Scientific, USA) (1:100 dilution in dimethylsulfoxide; Sigma, Germany), vortex-mixed and incubated at 37 °C in the dark for at least 15 minutes before measurement.
Instrument	Attune NxT flow cytometry (ThermoFisher Scientific) equipped with a blue laser set at 50 mW and tuned at an excitation wavelength of 488 nm.
Software	All data were statistically analyzed with the Attune NxT flow cytometry software.
Cell population abundance	For each processed sample, a non-stained aliquot was processed with flow cytometry to define the background noise.
Gating strategy	Cell debris was excluded from acquisition analysis by setting a BL1 threshold. Furthermore, the gated fluorescence events were evaluated on the forward-sideways density plot to exclude remaining background events and to obtain an accurate microbial cell count.

☐ Tick this box to confirm that a figure exemplifying the gating strategy is provided in the Supplementary Information.
